# Impaired Subset Progression and Polyfunctionality of T Cells in Mice Exposed to Methamphetamine during Chronic LCMV Infection

**DOI:** 10.1371/journal.pone.0164966

**Published:** 2016-10-19

**Authors:** Uma Sriram, Beth L. Hill, Jonathan M. Cenna, Larisa Gofman, Nicole C. Fernandes, Bijayesh Haldar, Raghava Potula

**Affiliations:** 1 Department of Pathology and Laboratory Medicine, Lewis Katz School of Medicine, Temple University, Philadelphia, United States of America; 2 Center for Substance Abuse Research, Lewis Katz School of Medicine, Temple University, Philadelphia, PA, United States of America; 3 Verity Software House, Topsham, Maine, United States of America; University of Missouri Kansas City, UNITED STATES

## Abstract

Methamphetamine (METH) is a widely used psychostimulant that severely impacts the host’s innate and adaptive immune systems and has profound immunological implications. T cells play a critical role in orchestrating immune responses. We have shown recently how chronic exposure to METH affects T cell activation using a murine model of lymphocytic choriomeningitis virus (LCMV) infection. Using the TriCOM (trinary state combinations) feature of GemStone^™^ to study the polyfunctionality of T cells, we have analyzed how METH affected the cytokine production pattern over the course of chronic LCMV infection. Furthermore, we have studied in detail the effects of METH on splenic T cell functions, such as cytokine production and degranulation, and how they regulate each other. We used the Probability State Modeling (PSM) program to visualize the differentiation of effector/memory T cell subsets during LCMV infection and analyze the effects of METH on T cell subset progression. We recently demonstrated that METH increased PD-1 expression on T cells during viral infection. In this study, we further analyzed the impact of PD-1 expression on T cell functional markers as well as its expression in the effector/memory subsets. Overall, our study indicates that analyzing polyfunctionality of T cells can provide additional insight into T cell effector functions. Analysis of T cell heterogeneity is important to highlight changes in the evolution of memory/effector functions during chronic viral infections. Our study also highlights the impact of METH on PD-1 expression and its consequences on T cell responses.

## Introduction

The prevention and treatment of chronic viral infections, such as HIV, present unique challenges due to the prevalence of a large population of patients that have chronic exposure to drugs of abuse [1]. Among these drugs of abuse, Methamphetamine (METH), a highly addictive stimulant seriously impacts management of chronic viral infections [2, 3], as evidenced by studies of various HIV-infected cohorts in the USA [4–6] and around the world [7, 8]. Much of the understanding of the adverse impact of stimulant use on immunological responses, in particular adaptive responses, has been gleaned from cross-sectional and longitudinal studies that have demonstrated mixed results. Some studies have shown no adverse effects on CD4/CD8 T cell parameters in HIV- positive (HIV+) or HIV-negative (HIV-) drug abusers [9] while other studies show a negative association [4, 10, 11]. Thus, the mechanisms by which chronic stimulant use perturb the adaptive immune system and susceptibility to opportunistic infections following chronic viral infections are still challenging to understand. The challenge is partly related to the existence of a complex and increasing number of T cell subsets with considerable heterogeneity in their functional capacity. Researchers have recently developed advanced software to carefully dissect out the T cell subsets without overlaps. We have used the Gemstone^®^ software (Verity Software House, Maine, USA) to analyze the polyfunctionality of T cells and discreetly study the progression of effector /memory T cells during the course of infection.

In this study, we used the classic viral model of chronic LCMV infection to study T cell responses [12, 13]. The following T cell functional markers were analyzed in the spleen: (1) the cytokines (IL-2, IFN-γ, TNF-α and TGF-β) which are representative of inflammatory/regulatory functions (2) the degranulation markers (perforin, granzyme B and CD107a) as representative of T cell cytotoxic functions and (3) CD44 and CD62L markers that classify T cells with respect to their memory/effector functions.

Our recent findings [14] indicate that the METH-induced microenvironment upregulates the expression of the immunoinhibitory programmed cell death-1 (PD-1) marker that is known to alter the homeostatic proliferation and differentiation pathways of T cell subsets [15–17], in an LCMV infection model. In this study, we analyzed correlations between PD-1 expression and T cell functions and report that METH-induced PD-1 upregulation altered the cytokine production as well as cytotoxic functions.

## Materials and Methods

### Mice

Male C57BL/6 mice (4 weeks of age) were purchased from Jackson Laboratory (Bar Harbor, ME, USA), housed in pathogen-free conditions, and given unlimited access to food and water. Protocols for the use of animals were in accordance with the guidelines and approval of the Institutional Animal Care and Use Committee of Temple University, which is an American Association for the Accreditation of Laboratory Animal Care accredited facility. All treated animals were monitored daily as part of the approved protocol. Mice were euthanized using carbon dioxide asphyxiation prior to tissue collection.

### METH treatment and LCMV infection

METH treatment and LCMV infection were performed as described [14]. Methamphamphetamine Hydrochloride was purchased from Sigma-Aldrich (St. Louis, MO). It has been shown that a good percentage of recreational METH abusers initially use lower doses and progressively increase the dose and eventually engage in increased amount and frequency of drug intake [18–20]. We used this rationale in this current study and to simulate a similar pattern, we used an escalating METH dose schedule. Mice were weight-matched and randomly divided into groups and were administered a gradual escalating METH dose from 0.45–10 mg/kg over 6 days, followed by a single dose of 10 mg/kg/day subcutaneously (s.c. at the nape of the neck) every day until the mice were sacrificed. Once a daily injection schedule of METH was established, mice were intravenously injected once with LCMV Clone 13 (2x10^6^ pfu). Peripheral blood was collected via submandibular vein into EDTA-coated collection tubes (BD Biosciences) every seven days until the end of the study. Mice were sacrificed at different time-points (days 14, 28 and 56) and spleens were collected and processed for flow-cytometric analysis. At least 3 mice were analyzed in each group for each time-point.

### Flow Cytometry

Splenocytes extracted from LCMV-infected mice were subjected to flow-cytometric analysis. After washing, splenocytes were resuspended in R10 medium containing LCMV peptide 69 (Proimmune, epitope of GP1, 1 μg/ml) and brefeldin A (eBioscience, 1 μl/ml). After incubation for 5–6 hrs at 37°C, cells were centrifuged, washed and stained with fluorescence-conjugated antibodies to cell surface markers and intracellular cytokines for flow cytometric analysis. Antibodies were purchased from eBioscience, San Diego, and BD Biosciences, CA, USA. The following antibodies were used for staining: CD3 APC-Cy7, CD4 AmCyan, CD8 Pacific Blue, CD44 PE, CD62L APC, CD279 (PD-1) PECy7, IFN-γ APC, IL-2 FITC, TNF-α PECy7, TGF-β PercpCy5.5, perforin FITC, granzyme B PECy7 and CD107a APC. Stained cells were then acquired on a FACS Canto II cytometer (BD Biosciences) using FACS Diva software (BD Biosciences, V 6.1.3). At least ten thousand lymphocyte events were recorded. The data was analyzed using Gemstone^™^ software (Gemstone^™^, Verity Software House, Maine, USA). The T lymphocyte population was first selected using CD3+ and further selected for CD4+ and CD8+ subsets. Data was further analyzed using TriCOM and Probability State Modeling in the Gemstone^™^ software.

### TriCOM analysis

**Fig 1:**
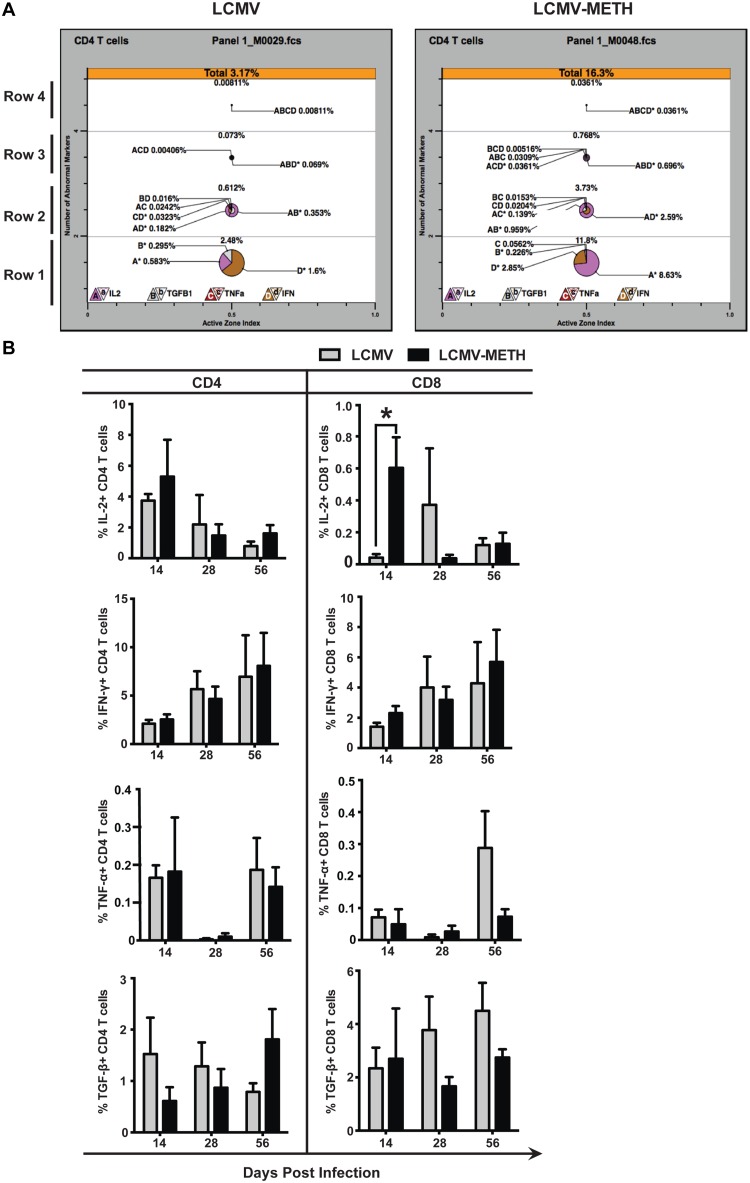
TriCOM analysis of cytokine expression modulated by METH in CD4 and CD8 splenocytes in chronic LCMV infection. **A**. Representative analysis of LCMV and LCMV-METH samples using TriCOM. Splenocytes were surface stained with CD3, CD4 and CD8 and intracellularly stained for IL-2, IFN-γ, TNF-α TGF-β cytokines. Bottom-most panel (row 1) shows percentages of each of the cytokines expressed in a cell. Rows 2, 3 and 4 show co-expression of two, three and four cytokines, respectively. **B**. Bar graphs showing percentages of CD4 (left panel) and CD8 (right panel) T cells positive for IL-2, IFN-γ, TNF-α and TGF-β, respectively.

**Fig 2:**
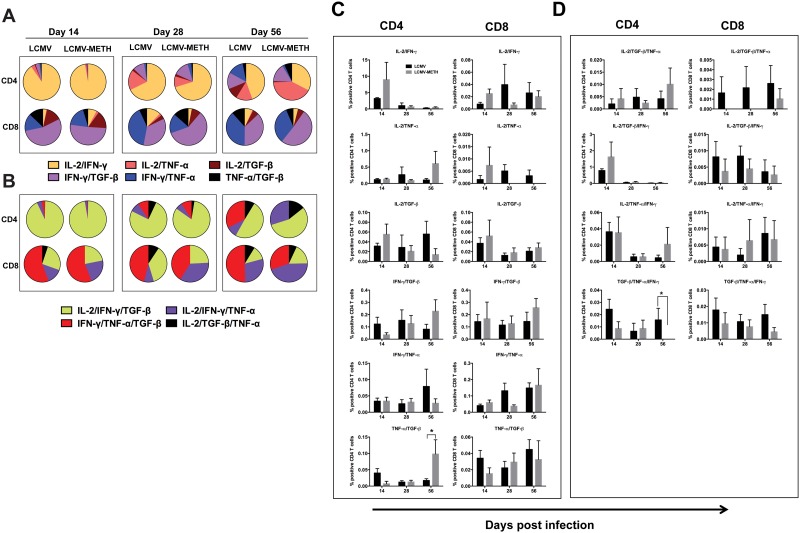
Analysis of co-expression of cytokines modulated by METH in CD4 and CD8 splenocytes in chronic LCMV infection. Pie charts show the changing patterns of cytokines of two (**A**) and three (**B**) co-expressed cytokines respectively, in CD4 and CD8 T cells in LCMV and LCMV-METH groups during chronic viral infection (days 14, 28 and 56). Numbers in the pie charts are averages of the percentages from TriCOM analysis. Bar graphs show the actual percentages of two (**C**) or three (**D**) co-expressed cytokines during LCMV infection.

TriCOM displays are a visualization of the polyfunctionality of T cells. Fig 1 shows a typical TriCOM representation. In Fig 1A, row 1 depicts the percent of cells expressing one cytokine, row 2 represents the cells co- expressing two cytokines, while rows 3 and 4 represent the cells co-expressing three or four cytokines. The key at the bottom shows how to interpret the phenotypes of the cytokine-producing cells. From these data we analyzed the percentage of cells positive for one cytokine (Fig 1B) or co-expression of two or three cytokine combinations, which are plotted as pie charts in Fig 2.

### Probability State Modeling

To characterize the phenotype of distinct T cell subpopulations, we adopted a novel approach based on Probability State Modeling (PSM) [21, 22] using Gemstone^™^ software, version 1.0.75. Based on this model, we identified four major CD4+ and CD8+ T cell subsets using three selection markers (CD3, CD4, CD8), side scatter (SSC) and a combination of CD62L and CD44 were used to stratify the subsets [23]. The subsets were defined as naïve (CD62L+ CD44-), central memory (T_CM_, CD62L+ CD44+), effector memory (T_EM_, CD62L- CD44+) and terminal effector (T_EF_, CD62L- CD44-). After the selection markers were set, CD62L was defined as a step-down (down-regulated), while CD44 was characterized as a 3-level expression profile, starting as negative in the naïve stage, up-regulating to positive and then down-regulating as the T cells matured. The stages were determined based on the phenotypes described by Inokuma et al. [22].

### Statistical analysis

Data were analyzed using Prism software version 6 (GraphPad, San Diego, CA). Correlation analysis was performed and *P* values were generated using Pearson correlation. The correlation “r” value and the *P* value are indicated in the Figs and tables. Student’s *t* test or one-way ANOVA, as appropriate, was used as a statistical test for comparison of groups and *P* < 0.05 was considered significant.

## Results

### METH exposure alters CD4 and CD8 T cell cytokine profile during chronic LCMV infection

Cytokine secretion (IFN-γ, TNF-α, and IL-2) by CD4 T cells is critical to sustain residual CD8 T-cell activity to control persistent infection [24, 25]. In this study, using TriCOM analysis, we analyzed the expression patterns of the cytokines IL-2, IFN-γ, TNF-α and TGF-β in the spleen (Fig 1A and 1B). TriCOM analysis provides an easy visual representation of markers expressed on cells (as seen in Fig 1A). The average values from the TriCOM percentages are presented in Fig 1B.

We previously reported that METH suppressed serum IL-2 levels in mice [14] and intracellular IL-2 levels in T cells isolated from human PBMC [26]. Interestingly, although intracellular IL-2 levels mirrored the serum levels at days 28 and 56-post infection, IL-2 expression was exacerbated in the LCMV-METH group compared to infection alone in the splenocytes of both CD4 and CD8 T cells at day 14 (LCMV vs. LCMV-METH—*P<0.05 for IL-2 expression in CD8 T cells). Contrary to the expression pattern of IFN-γ of LCMV mice in circulation as previously reported [14], in the current study the intracellular IFN-γ expression in the splenocytes increased after day 14 and remained stable up to day 56 both in CD4 and CD8 cells. METH did not modulate IFN-γ expression in the splenic T cells. The trend of intracellular expression of TNF-α was similar in the splenic CD4 T cells and mirrored that observed in the serum (in circulation). The expression pattern was bimodal with increased TNF-α expression by day 56 in both CD4 and CD 8 T cells (Fig 1B). Mice exposed to chronic METH could not increase TNF-α expression as compared to the LCMV infection alone at day 56.

Expression of TGF-β over the course of infection was relatively stable in CD4 and the CD8 T cells in the LCMV and LCMV-METH groups. METH decreased TGF-β expression on CD4 T cells at day 14 and increased by day 56. METH decreased TGF-β expression on CD8 T cells at days 28 and 56. Although these trends were striking, none reached statistical significance (Fig 1B).

### METH alters T cell intracellular cytokine co-expression patterns during chronic LCMV infection

T cells are one of the most diverse immune cell types. Analysis of polyfunctionality of T cells is critical for understanding the functional role of T cells in immune responses and assessing the status of ongoing T cell-mediated immune responses. The importance of these kinds of analyses has come to light in recent years in the context of infection [27–30]. In this report, we used TriCOM analysis to study polyfunctional T cell profiles in CD4 and CD8 populations in the spleen during chronic infection and changes brought about by METH treatment.

We averaged the percentages of expression of two (Fig 2A and 2C) or three cytokines (Fig 2B and 2D) in CD4 and CD8 T cells, during the course of LCMV infection. There were remarkable changes in the profiles of cytokines between the CD4 and the CD8 subsets. There was an increased induction of CD4 T cells co-expressing IL-2/IFN-γ compared to CD8 T cells (Fig 2A, yellow). IL-2-/IFN-γ was the only major cytokine combination at day 14 in the CD4 T cells (Fig 2A). IL-2-/IFN-γ co-expressing CD4 T cells decreased during the course of infection. While METH increased the percentage of both CD4 and T cells co-expressing these cytokines, the results did not reach statistical significance.

Only a small percentage of both CD4 and CD8 T cells co-expressed IL-2/TNF-α (Fig 2A, peach) compared to other cytokine combinations. The actual percentage of CD4 T cells co-expressing these cytokines was higher than in CD8 T cells (Fig 2C). The expression remained stable during the course of infection in CD4 T cells, while METH decreased the expression as the infection proceeded in the CD8 T cells.

The actual percentages (Fig 2C) of IL-2/TGF-β co-expressing CD4 and CD8 T cells were similar (Fig 2A, dark brown). Exposure to METH increased the expression at day 14 in both the CD4 and CD8 T cells. METH strongly decreased this expression combination in the CD4 T cells at day 56, while METH did not affect the expression in the CD8 T cells.

IFN-γ /TGF-β co-expression was again similar as actual percentages in the CD4 and CD8 T cells (Fig 2C). When compared to the other cytokine combinations, IFN-γ /TGF-β (Fig 2A, purple) was much higher in the CD8 T cells than in the CD4, that was dominated by IL-2/IFN-γ expression (Fig 2A, yellow).

IFN-γ/TNF-α co-expression (Fig 2A, blue) was higher in the CD8 T cells than in the CD4 T cells (Fig 2A and 2C). METH did not alter the expression of this combination in the CD4 T cells. There was a decrease in expression in the CD8 T cells with METH exposure at day 28 only (Fig 2A and 2C).

TNF-α/TGF-β co-expression (Fig 2A, black) was lower in the CD8 T cells compared to the CD4 T cells (Fig 2C), but this combination was slightly higher in the CD8 T cells than IL-2/TNF-α or IL-2/TGF-β. A slight decrease was observed with METH treatment at day 14 but TNF-α/TGF-β co-expression significantly increased at day 56 in the CD4 T cells (Fig 2C, *P<0.05).

Analysis of three cytokine combinations (IL-2/IFN-γ/TGF-β, IL-2/IFN-γ/ TNF-α, IL-2/TGF-β/TNF-α and IFN-γ/TNF-α/TGF-β) again revealed very different patterns in expression between the CD4 and the CD8 T cells (Fig 2B). IL-2/IFN-γ/TGF-β (Fig 2B, light green) was the most predominant cytokine combination when compared to the others in the CD4 T cells, especially at day 14. The expression of this combination decreased over time (Fig 2D). METH slightly increased the percentage of CD4 T cells expressing this combination. A considerable percentage of CD8 T cells also co-expressed IL-2/IFN-γ/TGF-β when compared to the other cytokine co-expression combinations. METH did not affect the percentage of CD8 T cells co-expressing IL-2/IFN-γ/TGF-β (Fig 2D).

IL-2/IFN-γ/TNF-α (Fig 2B, dark purple) co-expressing CD4 T cells started to populate as the LCMV infection proceeded and the IL-2/IFN-γ/TGF-β co-expressing cells began to decline in numbers. However, the actual percentages decreased from day 14 to day 56 in the CD4 T cells (Fig 2D). IL-2/IFN-γ/TNF-α co-expression remained quite stable during the entire course of infection (from day 14 to day 56). METH did not alter the expression pattern of IL-2/IFN-γ/TNF-α in the CD8 T cells during the entire course of infection.

CD4 T cells co-expressed the IL-2/TGF-β/TNF-α combination slightly more than the CD8 T cells (Fig 2D). METH increased the expression in CD4 T cells at day 56 (Fig 2B, black). IL-2/TGF-β/TNF-α co-expression was totally absent in the presence of METH at days 14 and 28 in the CD8 T cells (Fig 2B and 2D).

The percentages of CD4 and CD8 T cells co-expressing IFN-γ/TNF-α/TGF-β were similar (Fig 2D). METH decreased the expression at day 14 and METH very significantly abrogated the IFN-γ/TNF-α/TGF-β co-expressing CD4 T cells at day 56 (Fig 2B, red and Fig 2D, *P<0.05). The pattern was similar in the CD8 T cells, but the numbers did not reach statistical significance.

Collectively, these data suggest that METH does affect the cytokine phenotype that might reflect on overall T cell function, including inflammation and cytotoxicity.

### Altered expression of T cell cytotoxic functional markers in mice exposed to METH during chronic LCMV infection

**Fig 3:**
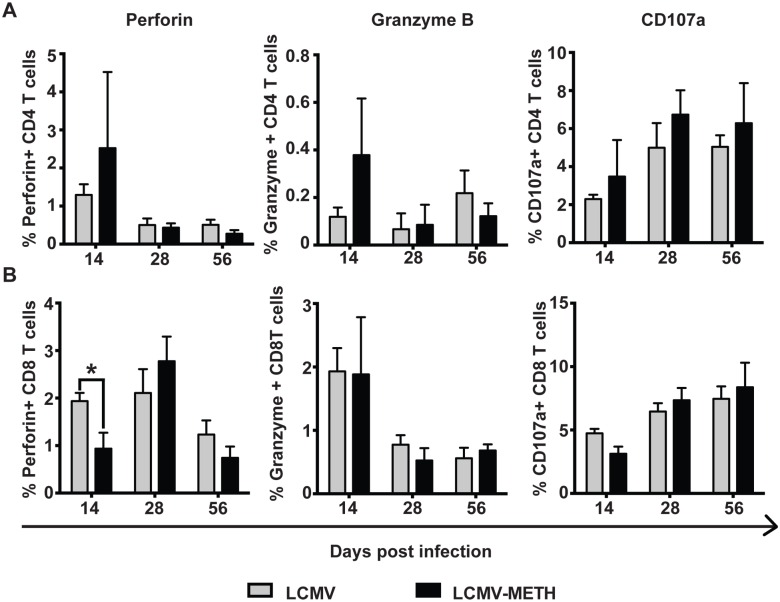
Expression of degranulation markers on CD4 and CD8 T cells in spleen in chronic LCMV infection. Bar charts represent the expression of perforin, granzyme B and CD107a degranulation markers in CD4 (**A**) and CD8 (**B**) T cells on days 14, 28 and 56 post LCMV infection, *P<0.05.

We analyzed the expression of perforin, granzyme B and CD107a as key markers to assess the cytotoxic functions of T cells. The percentage of CD8 T cells expressing perforin was higher than in CD4 T cells irrespective of the groups (Fig 3A and 3B). METH significantly decreased perforin expression at day 14 in the CD8 T cells (Fig 3B, *P<0.05). There was an increase in perforin expression at day 28 in the METH-exposed mice, that decreased again at day 56.

Regardless of the groups, a greater percentage of CD8+ T cells expressed granzyme B than did CD4+ T cells. Granzyme B expression gradually decreased as the infection proceeded. METH did not alter granzyme B levels in CD8 T cells (Fig 3B).

CD107a expression was similar in CD4 and CD8 T cells, unlike perforin and granzyme B. CD107a slightly increased in expression in CD4 and CD8 T cells over the course of infection. METH slightly increased CD107a expression on CD4 T cells (Fig 3A). These data suggest that METH alters the cytotoxic functions of T cells by altering the expression of degranulation markers.

### METH-induced cytokines affect cytotoxic functional markers’ expression in T cells during chronic LCMV infection

**Fig 4:**
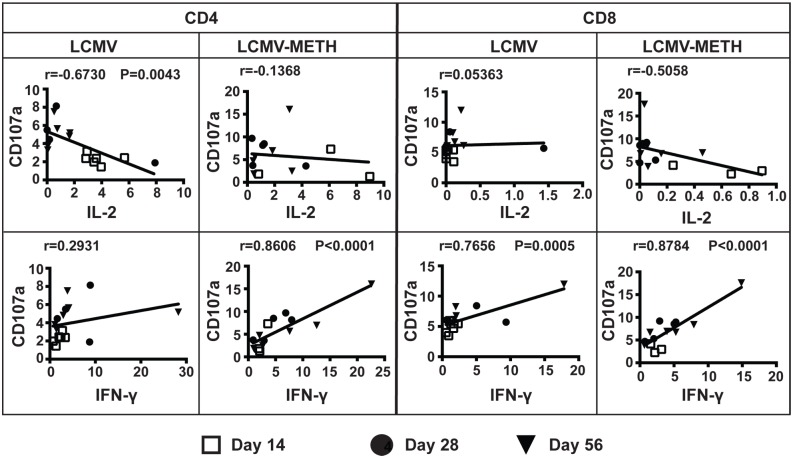
Correlation analysis between cytokine and degranulation marker expression in CD4 and CD8 T cells in chronic LCMV infection. Plots represent correlation analysis of IL-2, IFN-γ and TNF-α cytokines and CD107a degranulation marker expression during chronic LCMV infection. The day 14, 28 and 56 time points are noted by different symbols. Pearson correlation value (r) and *P* value (if any) are indicated in each plot.

We further extended the analysis to determine if there was any correlation between cytokine production and degranulation following METH treatment. As seen in the correlation plots (Fig 4), the expression of CD107a was strongly negatively correlated with IL-2 and IFN-γ production in CD4 T cells. Chronic exposure to METH during the course of infection disrupted this correlation. The correlation effects were quite opposite for CD107a and IL-2 in CD8 T cells. METH actually increased the linear trend of both decreased IL-2 and CD107a expression during the course of infection (Fig 4).

Exposure to METH during chronic LCMV infection showed a strong positive correlation with increased IFN-γ and a corresponding increase in CD107a expression on CD4 T cells. There was no correlation between IFN-γ and CD107a with infection alone in the CD4 T cells (Fig 4). In CD8 T cells, there was a positive correlation between IFN-γ and CD107a that was enhanced in the presence of METH.

The correlations between cytokines (IL-2 and IFN-γ, TNF-α and TGF-β) and degranulation markers (perforin, granzyme B and CD107a) are shown in Table 1. There was no correlation between IL-2 and perforin expression on CD4 T cells during LCMV infection alone. However, exposure to METH increased the correlation in a positive manner. IL-2 negatively correlated with perforin expression in CD8 T cells. IFN-γ negatively correlated with perforin expression in both CD4 and CD8 T cells. METH exposure induced a strong positive correlation (*P<0.05) between TNF-α and perforin expression in CD4 T cells compared to LCMV infection alone; however the expression remained inversely correlated in the CD8 T cells in both LCMV and LCMV-METH groups. TGF-β was strongly positively correlated with perforin expression (**P<0.001) in the CD4 T cells; METH altered this to a negative correlation. TGF-β was negatively correlated with perforin expression in CD8 T cells, which was unaltered by METH.

**Table 1:** Cytokines and degranulation marker correlations. Numbers in table represent the Pearson correlation r values.

Cell type	Cytokine	Treatment	Perforin	Granzyme B	CD107a
CD4	IL-2	LCMV	0.0162	-0.2375	-0.6730**
		LCMV-METH	0.4075	0.2586	-0.1368
	IFN-γ	LCMV	-0.4101	0.0508	0.2931
		LCMV-METH	-0.1004	-0.3022	0.8606***
	TNF-α	LCMV	0.0567	0.3321	-0.0551
		LCMV-METH	0.6570*	0.5917*	-0.0116
	TGF-β	LCMV	0.6465**	-0.448	-0.0101
		LCMV-METH	-0.0978	-0.215	-0.0215
CD8	IL-2	LCMV	-0.1282	-0.1268	0.0536
		LCMV-METH	-0.4093	0.1887	-0.5058
	IFN-γ	LCMV	-0.3675	-0.2156	0.7656***
		LCMV-METH	-0.2523	-0.1241	0.8784***
	TNF-α	LCMV	-0.3358	-0.0613	0.3825
		LCMV-METH	-0.2108	0.4882	0.1701
	TGF-β	LCMV	-0.2112	-0.3093	0.0841
		LCMV-METH	-0.3404	-0.094	0.0872

*P<0.05;

**P<0.001;

***P<0.0001

Similar to the positive IL-2-perforin correlation, the IL-2-granzyme B correlation was also altered in a positive manner both in CD4 and CD8 T cells. METH shifted the IFN-γ–granzyme B correlation to a negative trend in the CD4 T cells; the CD8 T cells remained negatively correlated for these two markers. TNF-α was positively correlated with granzyme B expression on CD4 T cells and METH strongly increased this correlation (*P<0.05). The correlation changed from negative to positive in CD8 T cells. TGF-β remained negatively correlated with granzyme B; METH did not change this trend in CD4 or CD8 T cells.

As shown in Fig 4 and Table 1, CD107a strongly negatively correlated with IL-2 in CD4 T cells. IFN-γ showed a strong positive correlation in both CD4 and CD8 T cells. While TNF-α was negatively correlated in CD4 T cells, CD8 T cells showed a positive trend. METH did not alter these patterns. Correlations with CD107a and TGF-β were very weak; CD4 T cells showed an inverse trend while CD8 T cells showed a direct linear trend. METH did not affect these trends. These data suggest that the cytokine production regulates the degranulation function and METH alters some of these effects.

### METH-induced PD1 upregulation impacts cytotoxic functional markers and cytokine expression in T cells during chronic LCMV infection

**Fig 5:**
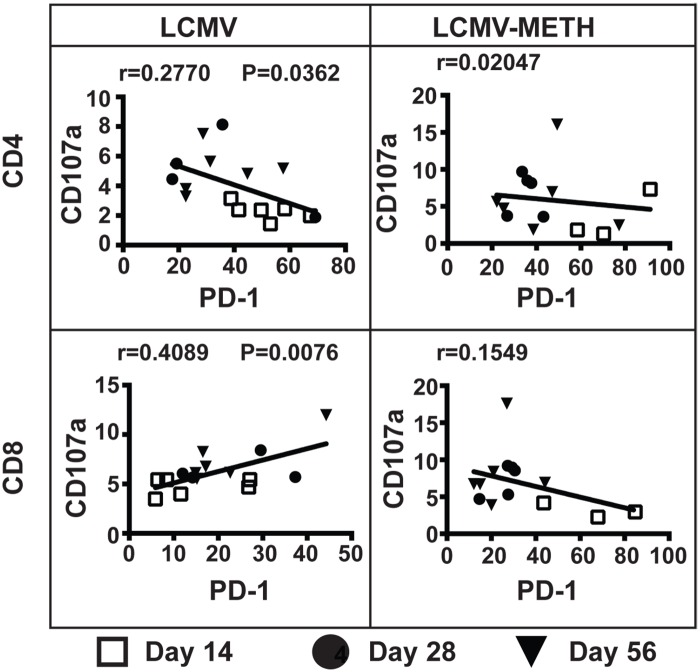
Correlation analysis between PD-1 and degranulation marker expression in CD4 and CD8 T cells in chronic LCMV infection. Plots represent correlation analysis of PD-1 expression and CD107a degranulation marker during chronic LCMV infection. The 14, 28 and 56 day time points are noted by different symbols. Pearson correlation value (r) and *P* value are indicated in each plot.

**Fig 6:**
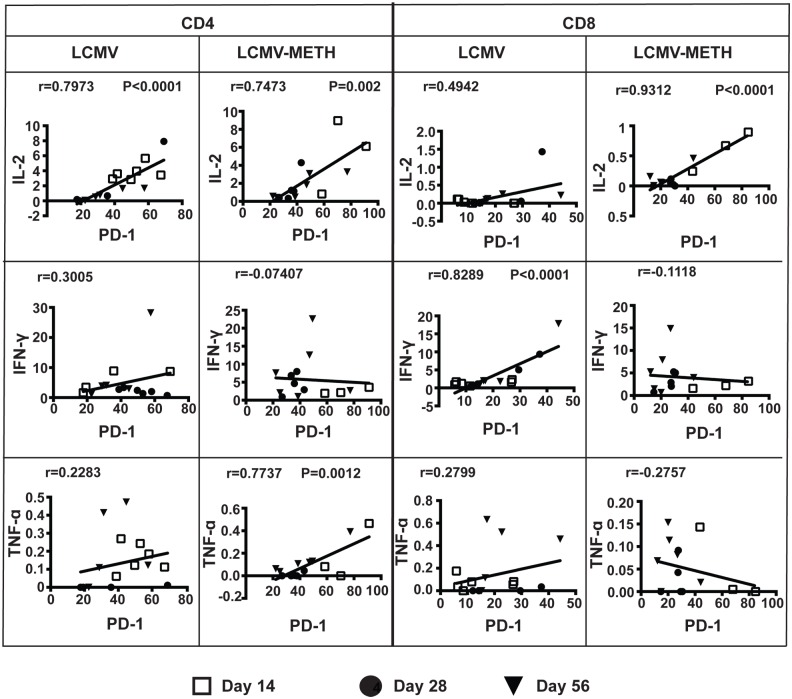
Correlation analysis between PD-1 and cytokine expression in CD4 and CD8 T cells in chronic LCMV infection. Plots represent correlation analysis of PD-1 expression with IL-2, IFN-γ or TNF-α cytokine expression during chronic LCMV infection. Day 14, 28 and 56 time points are noted by different symbols. Pearson correlation value (r) and *P* value (if any) are indicated in each plot.

We showed in our recent studies that METH increased the PD-1 inhibitory marker on CD4 and CD8 cells in the LCMV chronic infection model [14]. In this study, we further analyzed if the expression of PD-1 correlated with the expression of cytotoxic functional markers (perforin, granzyme B and CD107a) (Fig 5, Table 2) and cytokine expression (IL-2, IFN-γ, TNF-α and TGF-β) (Fig 6).

**Table 2:** PD-1 –degranulation marker correlations. Numbers in table are Pearson correlation r value.

Cell type	Treatment	Marker	Perforin	Granzyme B
CD4	LCMV	PD-1	-0.0759	-0.7973
	LCMV-METH	PD-1	0.6032*	0.4916
CD8	LCMV	PD-1	-0.0961	-0.1042
	LCMV-METH	PD-1	-0.1791	0.3129

*P<0.05

In LCMV-infected mice we found no correlation between PD-1 and perforin expression with infection, but in infected mice exposed to METH there was a trend towards a strong positive correlation (Table 2, *P < 0.05) of increased perforin expression with increasing PD-1 expression on CD4 T cells.

The CD8 T cells remained negatively correlated between PD-1 and perforin, while METH did not change this trend. Of note, similar to perforin, the expression of granzyme did not correlate well with PD-1 expression in the infected mice, while METH altered it to a positive trend in both CD4 and CD8 T cells (Table 2). Interestingly, CD107a expression followed a negative correlation in CD4 T cells (*P < 0.05) and a strong positive correlation in CD8 T cells (**P < 0.001) with LCMV infection (Fig 5). These correlations were altered upon METH treatment (Fig 5).

We also analyzed the correlation between PD-1 and cytokine expression in CD4 and CD8 T cells and observed a strong positive correlation between PD-1 and IL-2 expression in CD4 as well as CD8 T cells. The trend was seen at all time points (Fig 6) both in the LCMV and LCMV-METH groups. We found no correlation between PD-1 and IFN-γ expression in CD4 T cells, whereas the strong positive correlation in the CD8 T cells upon infection (***P<0.0001) was altered to a negative trend with METH treatment. METH increased the correlation pattern of TNF-α with PD-1 in the CD4 T cells but not CD8 T cells. An inverse trend of TGF-β vs. PD-1 expression was observed with infection, which was altered to a positive correlation upon METH exposure in CD4 and CD8 T cells (CD4—LCMV vs. LCMV-METH–r = -0.0965 vs. r = 0.1949; CD8—LCMV vs. LCMV-METH–r = -0.06738 vs. 0.3093). These analyses revealed that, during chronic LCMV infection, upregulation of PD-1 induced by METH modulates T cell cytotoxic function.

### Probability State Modeling analysis of METH-induced changes in T cell effector/memory differentiation during chronic LCMV infection

**Fig 7:**
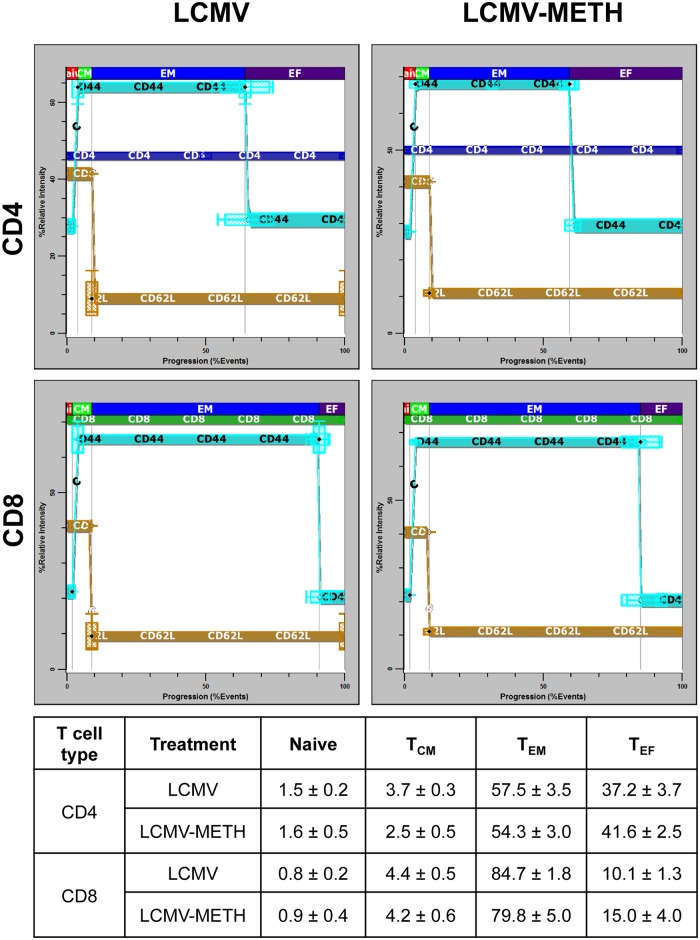
Probability State Modeling of T cell progression modulated by METH in chronic LCMV infection. Average Probability State Model (PSM) progression plots of CD4+ (top panel) and CD8+ (bottom panel) T cells from spleens from LCMV (n = 6) and LCMV-METH (n = 3) treated mice (14 days post-infection). The subsets are defined by the correlated measurements CD3, SSC (side-scatter), CD8, CD4, CD62L and CD44. The x-axis represents CD4 or CD8 T-cell antigen-dependent progression with units of cumulative percentage of events. The y-axis is the relative dynamic range of the measurement intensities between 0 and 100. The end of the naïve stage (red) is defined as the beginning of the up-regulation of CD44 (first black diamond). The end of the CM (green) stage is defined by the down-regulation of CD62L (black diamond), while the end of the EM stage (blue) and the beginning of the EF stage (brown) are the end of the down-regulation of CD44 (second black diamond). Each controlled definition point (CDP) in an expression profile (EP) is shown as a white or black diamond. Each CDP in the progression plot has a vertical box whisker for examining the variability of measurement intensities and a horizontal box whisker for examining the variability of associated cumulative percentages. Table shows the mean ± SEM of the percentage of the T cell subsets in the CD4 and CD8 cell types at day 14.

During infection, T cells differentiate into multiple types of effector and memory T cells, which mediate pathogen clearance and provide long-term protective immunity [31, 32]. To characterize the phenotype of distinct T cell subpopulations in our LCMV model, we adopted a novel approach based on the Probability State Modeling (PSM) system to identify and quantify subsets and to avoid subjective gating and errors associated with a conventional gating approach for analysis of multi-parameter flow-cytometric data. A model was generated using Gemstone software, version 1.0.75 [22], to analyze the progression of the markers, as described in the Materials and Methods section. Peptide-stimulated splenocytes were stained for CD3, CD4, CD8, CD62L and CD44 markers. Stained samples from LCMV (n = 6) and LCMV-METH (n = 3) treated mice were acquired in a BD FACS Canto II flow cytometer. Data from each group of mice were modeled and averaged. The results are summarized in Fig 7 for the day 14 time-point. Each controlled definition point (CDP) in the progression plot has a vertical box whisker for examining the variability of measurement intensities and a horizontal box whisker for examining the variability of cumulative percentages. Numbers in the table show the percentages (mean ± SEM) of the CD4 and CD8 T cell subsets. The percentages of T_EM_ decreased and T_EF_ increased by day 28 and 56 in CD4 and CD8 T cell populations in both LCMV and LCMV-METH study groups (S1 Fig). The plots show some differences in percentages of the different T cell subsets but no significant changes between the LCMV and LCMV-METH groups, suggesting that METH did not impact T cell subset progression in our data set (Fig 7).

**Fig 8:**
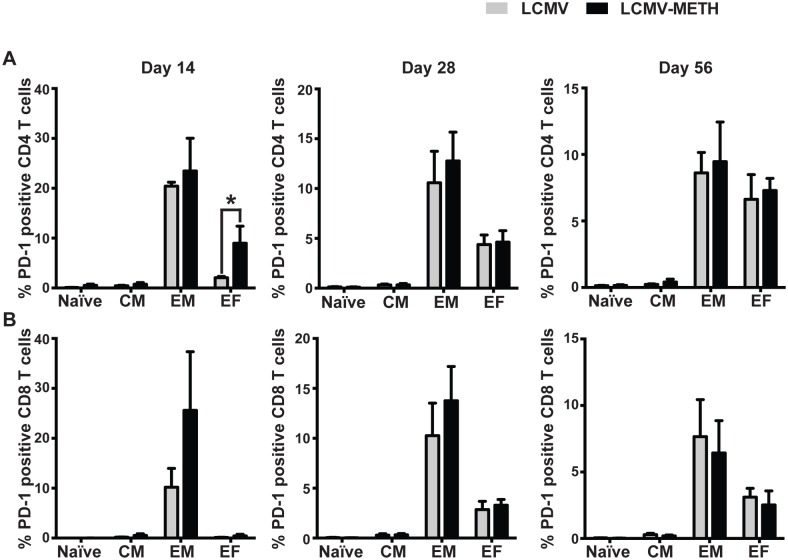
PD-1 expression in T cell subsets in the spleen. Percentages of PD-1 expression in CD4 (**A**) and CD8 (**B**) subsets from the PSM analysis, Data are presented as mean ± SEM, *P<0.05.

We have shown recently that METH increased PD-1 expression on CD4 and CD8 T cells during chronic LCMV infection [14]. We further extended our analysis in this study to measure PD-1 expression in the T cell effector/memory subsets by PSM. Percentages of PD-1 expressing subsets were measured and analyzed over time (days 14, 28 and 56) during chronic LCMV infection (Fig 8). A trend towards increased PD-1 expression was observed at days 14 and 28 in the METH-treated LCMV-infected group compared to LCMV alone in the CD8 effector memory (T_EM_) subset. PD-1 was significantly increased in the terminal effector (T_EF_) CD4 subsets at day 14 (Fig 8, *P<0.05); however, these differences did not reach significance at later time-points. In chronic infection models, an exhausted phenotype is exhibited because of continuous expression of viral antigen that leads to dysfunctional CD8 cells. Importantly, these cells express several inhibitory receptors including PD-1 [33]. Sauce et al. have shown that PD-1 expression on CD8 T cells, including HIV-specific cells, is linked to the stage of T cell differentiation [34]. Reduction in the expression of inhibitory receptors is crucial for the formation of optimal CD8 memory cells [33]. Our results indicate that an increase in PD-1 expression in T cell subsets might alter memory responses during LCMV infection.

## Discussion

Acute or chronic viral infections can lead to generalized immunosuppression. Several mechanisms, such as immunopathology of CD8+ T cells, inhibitory receptors, or regulatory T cells, contribute to immune dysfunction. The availability of advanced analysis tools has made it possible to dissect out the polyfunctionality of individual T cell subsets to enhance our knowledge about the quality and heterogeneity of immune response [27, 29, 35, 36]. Changes in the patterns of various markers co-expressed during the course of infection aid in better understanding of the disease course for treatment intervention [37]. In this study, we have used TriCOM analysis to analyze patterns of cytokine profiles in CD4 and CD8 T cell populations and provide an overview of how METH affected these patterns during chronic LCMV infection. We observed distinct patterns in cytokine co-expression in CD4 and CD8 subsets (Fig 2). METH exposure altered the co-expression IFN-γ/TNF-α/TGF-β, the key cytokines produced during LCMV infection. Co-expression of IFN-γ/TNF-α/TGF-β was significantly affected by METH in the CD4 T cell subpopulation that was very dramatic at the last time point (day 56) of infection. An earlier report showed that METH stimulates secretion of TNF-α in the splenocytes of retrovirus-infected mice [38]. METH has been shown to decrease CD4 T cell frequency and alter pro-inflammatory cytokine production in a self-administration model of drug abuse [39]. In that study, the METH lifetime dose was inversely correlated with serum TNF-α levels, while another study showed that TNF-α was increased in splenocytes with METH treatment [38]. TGF-β is an immunosuppressive cytokine known to impede both self- and tumor-specific T cells, but its role in regulating antiviral immunity is not entirely understood. Zinkernagal and colleagues have shown that TGF-β impaired the immune-mediated elimination of LCMV and delayed and impaired the LCMV-triggered CTL-mediated immunopathologic disease [40]. Our study reveals that METH increased TGF-β expression on CD4 T cells at day 56 (Fig 1B) in contrast to a decrease in the TGF-β expression on CD8 T cells at later time points (Fig 1B). This trend is in line with our observation that METH decreased IL-10 expression, another regulatory cytokine, in circulation [14]. The co-expression of IL-2/TGF-β/TNF-α is decreased by METH in CD8 T cells, suggesting that METH deregulates the immunoregulatory capacity of T cells. The role of these regulatory cytokines in the context of METH is still unclear. The mechanisms by which METH regulates suppressive/regulatory cytokines such as TGF-β may be important to pursue in future investigations.

Among the markers that we analyzed for cytotoxic function, we found that METH strongly affected the correlations between cytokines and the degranulation marker CD107a (Fig 4). However, METH did not affect the expression of perforin or granzyme B in our analysis, either as independent expression on T cells or regulated by cytokines as shown by correlation statistics. The expression of CD107a is emerging as an important marker of cytotoxic functions during chronic infections [41]. An HIV study cohort reveals that ART-naïve individuals had a significantly greater CD107a response compared with treated individuals, independent of when treatment was initiated [37]. In addition, the co-expression of CD107a and IFN-γ was significantly higher in ART-naïve individuals compared with treated individuals [37]. METH modulation of the expression of CD107a, either directly or by affecting other markers such as cytokines that might influence CD107a expression, may have clinical relevance for understanding the pathogenesis during chronic viral infections.

A successful immune response against chronic viral infections depends on virus-specific CD8+ T cells. However, during chronic infection, these CD8 T cells are impaired in their antiviral function or undergo functional inactivation (also known as exhaustion). Expression of PD-1 has been linked to decreased proliferative capacity in CD8 T cells [42]. The functional exhaustion of CD8 T cells has been shown to correlate with expression of multiple inhibitory receptors including PD-1 [16, 17, 43, 44]. Importantly, we have shown that METH significantly upregulated PD-1 on both CD4 and CD8 T cells in our chronic LCMV model [14]. In a recent study using a cohort of hepatitis B virus (HBV) infected patients, HBV-specific CD8 T cells showed a negative correlation between PD-1 expression and cytokines and degranulation markers. Increased PD-1 expression and decreased IFN-γ, TNF-α, and CD107a expression was observed in these antigen-specific CD8 T cells [45]. Our analysis of PD-1 expression in relation to cytotoxic markers in our LCMV model showed that PD-1 expression had a significant negative correlation with CD107a marker in the CD4 subset, but positively correlated in the CD8 subset (Fig 5). There was a strong positive correlation of IFN-γ with PD-1 expression in our model (Fig 6).

Crucial elements of T cell-mediated immunity include a primary response by naïve T cells, effector functions by activated T cells, and persistence of antigen-specific memory T cells [46, 47]. Studies have shown individual CD8+ T cells exhibit complex, combinatorial cytokine expression phenotypes and exist as distinct cell states (e.g., central memory or terminal effector) [30, 31]. Understanding the heterogeneity of the T cell populations has been a challenge to investigators in this field. With the development of new approaches for the analysis of multi-parameter flow data, in this report we could identify and quantify subsets. We classified events into populations by probability based on a model defined by basic biological information (Probability State Model). Using this method, subjective gating and associated errors are eliminated.

A cardinal feature of adaptive immunity is immunological memory. Understanding the adverse effect of METH use on the diverse T lymphocyte subsets that provide acute and long-term protection from infection is an important goal. Early during chronic infection, the fate of virus-specific CD8 T cells remains plastic, while later exhausted CD8 T cells become fixed in their differentiation state. Moreover, exhausted CD8 T cells arise from the memory precursor and not the terminally differentiated subset of effector CD8 T cells [35, 48]. We did not see a huge impact of METH exposure on the development of memory/effector T cells either in the CD4 or the CD8 T cell compartments in our LCMV model. However, analysis of functional markers such as PD-1 in the different subsets revealed that METH increased PD-1 expression in the effector subsets (T_EF_) in the CD4 compartment (Fig 8). We also observed a trend in increased PD-1 expression in the CD8 T_EM_ subset of METH treated mice than LCMV alone at days 14 and 28.

Memory CD8 T cells have the key ability to reactivate multiple effector functions and undergo vigorous proliferation upon antigen re-exposure [49]. In contrast to the high functional capacity of effector and memory CD8 T cells generated after acute infection or vaccination, CD8 T cell function is often impaired or exhausted during chronic infections. CD8 T cell exhaustion appears to be a prominent feature not only of experimental chronic infections in mice but also of chronic infections in primates and humans [15]. Exhausted CD8 T cells also fail to acquire memory T cell properties, such as antigen-independent self-renewal and the ability to mount robust recall responses [50]. Wherry and Kurachi [51] have recently described a distinct T exhausted subset (T_EX_) that is high in PD-1 expression. The development of T_EX_ remains incompletely understood. Persistent antigen stimulation appears to be a key signal driving exhaustion. Other types of signals such as proinflammatory and suppressive cytokines, other regulatory leukocytes, and components of the tissue microenvironment such as pH seem to be important for the generation of the T exhaustion phenotype. In conjunction with our own observation of METH effects on the expression of PD-1 on effector/memory T cells, it is possible that METH may have a very important effect on the generation of the newly-defined T_EX_ subset. Blocking of PD-1 expression/signaling has been the goal of many investigators who have been studying chronic viral infections and cancer [52, 53]. More extensive studies on the effects of METH on PD-1 expression and modulation of T cell functions, as well as the impact on PD-1 blockade are very important to provide insights for better vaccination and treatment strategies.

## Supporting Information

S1 Fig PSM analysis of CD4 and CD8 T cell subsets. The percentage of the different subsets analyzed by Probability State Modeling (PSM) is represented as bar graphs. Data are mean ± SE of LCMV and LCMV-METH groups. Upper panel shows CD4 subsets and lower panel shows CD8 subsets at days 14, 28 and 56, post infection.
(DOCX)
