# Supplementary material for: Impaired Subset Progression and Polyfunctionality of T Cells in Mice Exposed to Methamphetamine during Chronic LCMV Infection
Source: PLoS One. 2016 Oct 19;11(10):e0164966. doi: 10.1371/journal.pone.0164966 (PMC5070876; doi:10.1371/journal.pone.0164966)
Supplement: S1 Fig — The percentage of the different subsets analyzed by Probability State Modeling (PSM) is represented as bar graphs. Data are mean ± SE of LCMV and LCMV-METH groups. Upper panel shows CD4 subsets and lower panel shows CD8 subsets at days 14, 28 and 56, post infection. (DOCX) [file pone.0164966.s001.docx]

**Supplemental Figure 1:**

The percentage of the different subsets analyzed by Probability State Modeling (PSM) is represented as bar graphs. Data are mean ± SE of LCMV and LCMV-METH groups. Upper panel shows CD4 subsets and lower panel shows CD8 subsets at days 14, 28 and 56, post infection.
